# Infective endocarditis causing mitral valve stenosis – a rare but deadly complication: a case report

**DOI:** 10.1186/s13256-017-1197-3

**Published:** 2017-02-17

**Authors:** Michael A. Hart, Gautam R. Shroff

**Affiliations:** 1 General Internal Medicine, Hennepin County Medical Center, 701 Park Avenue, Minneapolis, MN 55415 USA; 2 Cardiology, Hennepin County Medical Center, 701 Park Avenue, Minneapolis, MN 55415 USA

**Keywords:** Case report, Infective endocarditis, Infectious disease, Echocardiography, Cardiovascular surgery

## Abstract

**Background:**

Infective endocarditis rarely causes mitral valve stenosis. When present, it has the potential to cause severe hemodynamic decompensation and death. There are only 15 reported cases in the literature of mitral prosthetic valve bacterial endocarditis causing stenosis by obstruction. This case is even more unusual due to the mechanism by which functional mitral stenosis occurred.

**Case presentation:**

We report a case of a 23-year-old white woman with a history of intravenous drug abuse who presented with acute heart failure. Transthoracic echocardiography failed to show valvular vegetation, but high clinical suspicion led to transesophageal imaging that demonstrated infiltrative prosthetic valve endocarditis causing severe mitral stenosis. Despite extensive efforts from a multidisciplinary team, she died as a result of her critical illness.

**Conclusions:**

The discussion of this case highlights endocarditis physiology, the notable absence of stenosis in modified Duke criteria, and the utility of transesophageal echocardiography in clinching a diagnosis. It advances our knowledge of how endocarditis manifests, and serves as a valuable lesson for clinicians treating similar patients who present with stenosis but no regurgitation on transthoracic imaging, as a decision to forego a transesophageal echocardiography could cause this serious complication of endocarditis to be missed.

**Electronic supplementary material:**

The online version of this article (doi:10.1186/s13256-017-1197-3) contains supplementary material, which is available to authorized users.

## Background

Infective endocarditis (IE) presenting as isolated valvular stenosis is a rare phenomenon; valvular regurgitation is seen much more commonly. The increasing accessibility of echocardiography has placed a premium on this particular component of the modified Duke criteria. However, IE-related valvular stenosis is notably absent from these criteria, and transthoracic echocardiography (TTE) may not provide enough sensitivity to reliably characterize valvular abnormalities. We present a case of rapidly progressive heart failure caused by prosthetic mitral valve stenosis in the setting of florid IE.

## Case presentation

A 23-year-old white woman presented to our hospital 8 months previously with methicillin-resistant *Staphylococcus aureus* (MRSA) sepsis and was found to have IE in the context of intravenous (IV) drug abuse. Transesophageal echocardiography (TEE) showed significant tricuspid and mitral valve vegetations with severe mitral regurgitation requiring salvage repair and reconstruction of both valves. She had a prolonged hospital stay but was successfully discharged with close cardiology follow up. Four months later, she developed heart failure in the context of medication noncompliance and underwent redo surgery with mitral valve replacement using a 25 mm St Jude Epic™ bioprosthesis. She was discharged home with prolonged antibiotic therapy and a plan to continue partaking in an extensive out-patient drug rehabilitation program.

Two months after her valve replacement, she was transferred to our facility from an outside hospital for suspected meningitis. On presentation, she stated that 1 week prior to admission she had had subjective fevers, headache, and myalgia, in the setting of recurrent IV drug use. She was febrile to 40.4 °C, with an initial blood pressure 90/60 mmHg, heart rate 147 beats per minute, and respiratory rate 32 breaths per minute. A cardiac examination showed normal S1 and S2 with no audible murmur and no notable jugular venous distension. Her lung sounds were clear bilaterally. An abdominal examination was unremarkable, and a skin survey demonstrated track marks over her left antecubital fossa with Janeway lesions over her fingertips. A neurologic examination was significant for a Glasgow Coma Scale (GCS) of 14 with altered sensorium, but no meningeal signs or focal deficits.

An initial workup yielded serum sodium of 127 mEq/L, potassium of 2.0 mEq/L, bicarbonate of 17 mEq/L with no anion gap, blood urea nitrogen of 28 mg/dL, and serum creatinine of 1.4 mg/dL believed to be secondary to hypovolemia from diarrhea. A complete blood count showed hemoglobin of 9.2 g/dL, platelets of 58 k/mm^3^, and no leukocytosis. Her anemia and thrombocytopenia were attributed to severe sepsis, with no evidence of disseminated intravascular coagulation. Her liver function tests were normal. Urine analysis showed moderate blood but no red blood cells, suggesting myoglobinuria. A urine drug screen was positive for methamphetamine and heroin. An electrocardiogram showed sinus tachycardia and known first-degree atrioventricular block. A chest X-ray was unremarkable, and computed tomography (CT) imaging was without evidence of acute intracranial pathology. A lumbar puncture showed glucose of 67 mg/dL, elevated protein at 57 mg/dL, neutrophilic pleocytosis with 217 white blood cells per cubic mm, and no evidence of organisms on Gram stain or culture.

**Fig. 1:**
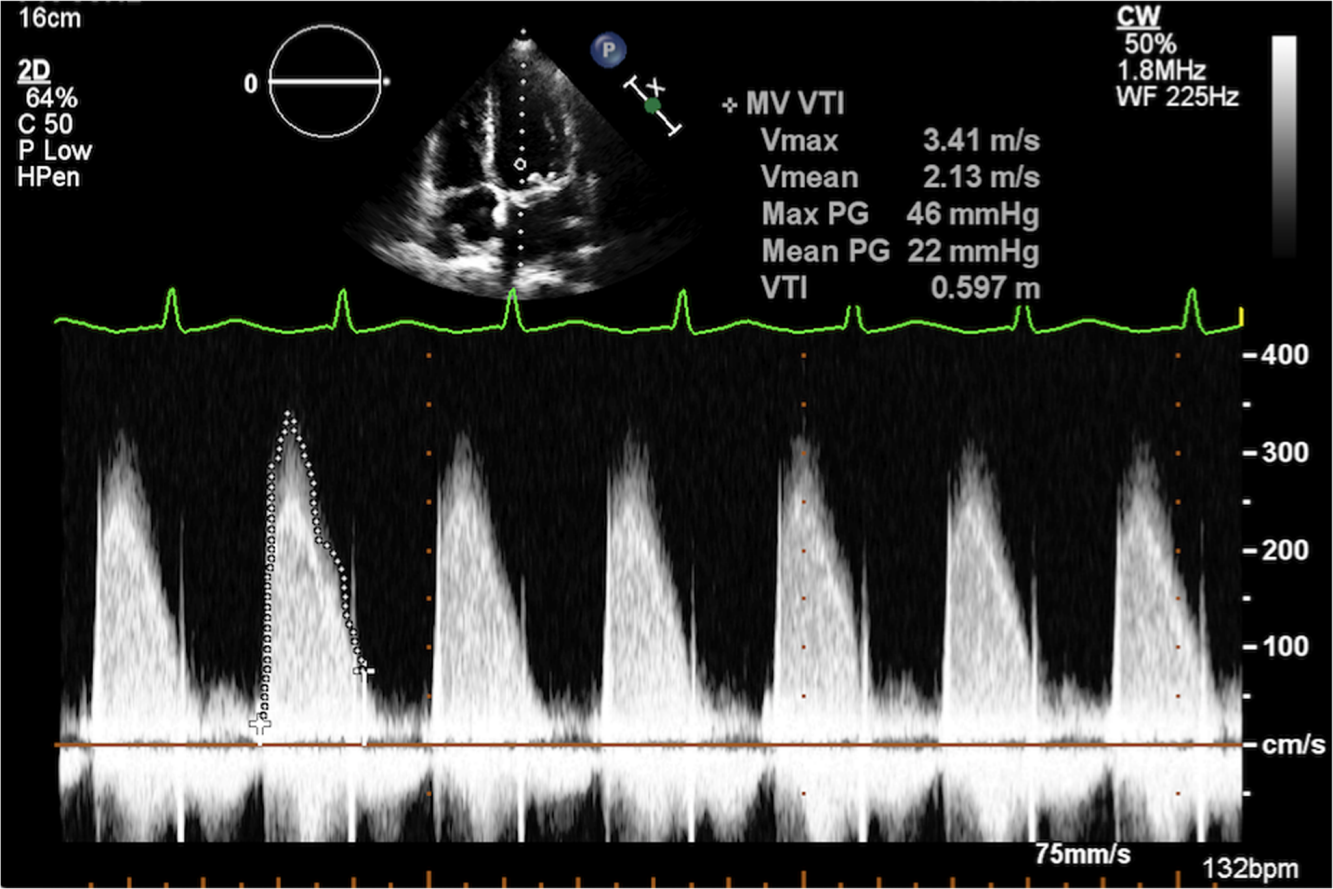
Doppler imaging by transthoracic echocardiography demonstrating a marked elevation in mean transmitral diastolic gradient of 22 mmHg at a heart rate of 132 beats per minute, indicating significant stenosis across the mitral prosthesis

**Fig. 2:**
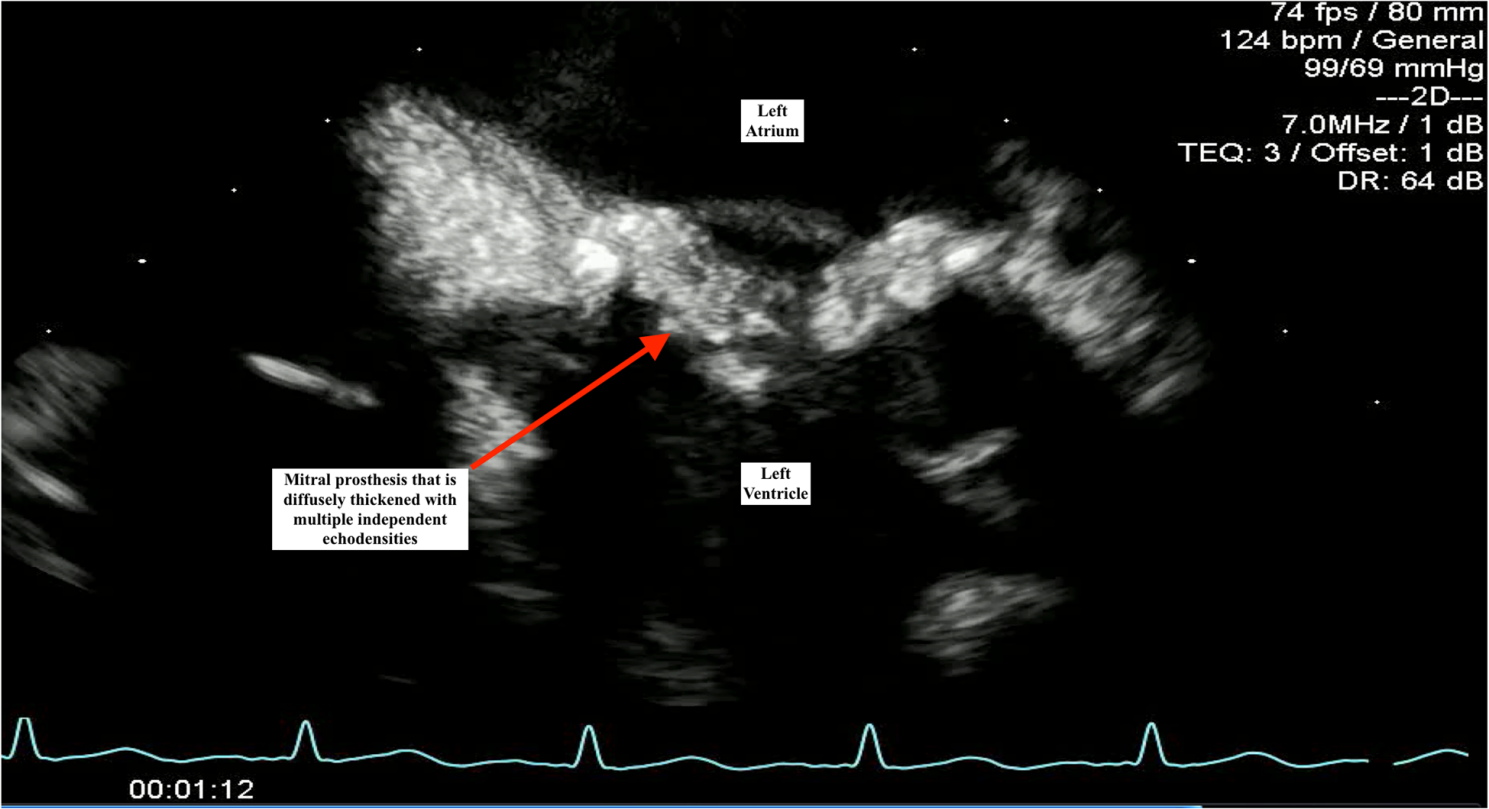
Transesophageal echocardiography showing multiple independent echodensities on the leaflets with significant soft tissue infiltration of the valve itself

**Fig. 3:**
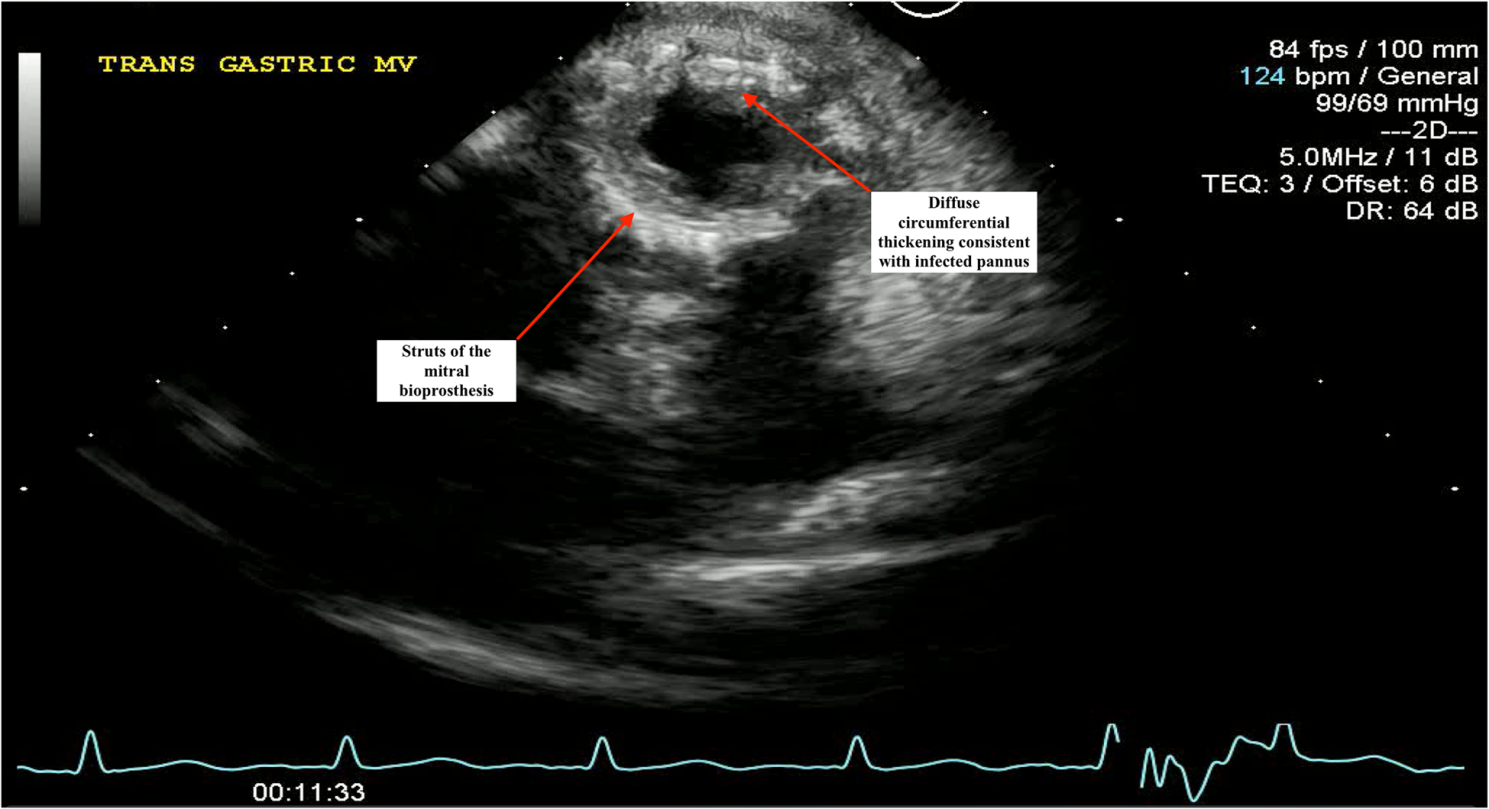
Transesophageal transgastric short axis view of the infected mitral bioprosthesis with diffuse thickening of the bioprosthetic leaflets and extensive infected pannus resulting in narrowing of the orifice, explaining the mitral stenosis

She was transferred to our intensive care unit; aggressive fluid resuscitation was administered intravenously for her hypotension and electrolyte abnormalities, and she continued on empiric antibiotics for suspected meningitis. Two sets of blood cultures grew methicillin-sensitive *Staphylococcus aureus* (MSSA), prompting a switch in therapy to nafcillin and gentamicin for presumed MSSA IE. Her gentamicin levels were subsequently followed during her treatment course to avoid worsening acute renal failure. TTE showed a mean mitral valve gradient of 22 mmHg indicating severe mitral stenosis, but notably failed to show any obvious vegetations or regurgitation (Fig. 1). Subsequent TEE re-demonstrated mitral stenosis, in addition to multiple independently mobile echodensities on the mitral prosthesis (Fig. 2). There was also evidence of significant thickening of the bioprosthetic leaflets with extensive infected pannus formation (Fig. 3). No mitral valve insufficiency was present (Additional files 1, 2 and 3).

Due to the extent of her endocarditis, it was very unlikely that medical therapy and supportive care alone would be sufficient treatment, and cardiothoracic surgery deemed her to be at prohibitive surgical risk given her ongoing drug abuse and poor prognosis. It was collectively decided to transition to palliative care given her persistent critical illness in the face of maximal medical therapy. She was kept comfortable until death a few days later.

## Discussion

The use of echocardiography in identifying IE was first described in 1973. Today, TEE is the gold standard for assessing suspected prosthetic valve endocarditis due to its superior sensitivity over traditional TTE [1, 2]. Clinicians use the modified Duke criteria for establishing a clinical diagnosis of definite or probable endocarditis [3]. Although valvular regurgitation or prosthetic valve dehiscence (which also causes regurgitation) is recognized as a major criterion to establish a diagnosis, stenotic lesions are not included. The pathophysiology of IE is more conducive to regurgitation, as vegetations tend to grow on the cusps themselves and prevent appropriate leaflet coaptation. Valvular destruction also contributes over time and may eventually induce complete flail leaflet, creating a nidus for turbulent regurgitant flow and further vegetation formation [1, 4]. Stenosis is much rarer and can be a complication of late or recurrent IE, but has also been described as a result of obstruction by large vegetations in the acute setting.

There have been a number of previously reported cases describing outflow obstruction from large bacterial vegetations on native valves, with the majority occurring in mitral valves with preexisting stenosis [5]. Approximately 15 cases have reported IE-related obstruction of a *prosthetic* valve, most often in the setting of fungemia [6]. Less represented in the literature are cases of pannus formation and infective infiltration of the valve itself. The case presented here is an example of the latter and is unusual in this regard. This infiltration causes functional inhibition of blood flow into the left ventricle, creating an increased diastolic gradient that can have deleterious hemodynamic effects by manifesting as pulmonary edema and heart failure [4, 7].

Historically, the outcomes for patients with IE-related stenosis have been poor given the extent of infection, but there have been more recent case reports of success with surgical intervention [4, 5, 7–10]. In light of this, identification of infection-related stenosis is critical, and clinicians should be cognizant that TTE can have significantly reduced sensitivity compared to TEE in detecting infective vegetations. Moreover, in the context of stenosis without regurgitation on TTE, a decision to forego a TEE could cause this serious complication of endocarditis to be missed.

## Conclusions

Mitral valve stenosis as a result of obstruction and/or impediment of proper valvular function is a rare but very serious complication of IE. In patients presenting with sepsis and new heart failure suspicious for endocarditis, it is crucial that a TEE is performed to better characterize valvular pathology. Modified Duke criteria should be used as a tool to help make this diagnosis, with the knowledge that valvular stenosis is not included in the echocardiographic criteria but can be a deadly manifestation of this syndrome.



**Additional file 2: Video S2.** Color Doppler illustrating a marked increase in the diastolic gradient consistent with severe mitral stenosis. (AVI 2117 kb)




**Additional file 3: Video S3.** Transesophageal transgastric short axis view of the infected mitral bioprosthesis with diffuse thickening of the bioprosthetic leaflets and extensive infected pannus resulting in narrowing of the orifice, explaining the mitral stenosis. (AVI 3416 kb)


## Additional files

Additional file 1: **Video S1.** Transesophageal echocardiography of the bioprosthetic mitral valve with evidence of multiple independently mobile echodensities on the leaflets and significant soft tissue infiltration of the valve itself. (AVI 733 kb)
